# miR-143 Targeting CUX1 to Regulate Proliferation of Dermal Papilla Cells in Hu Sheep

**DOI:** 10.3390/genes12122017

**Published:** 2021-12-18

**Authors:** Tingyan Hu, Sainan Huang, Xiaoyang Lv, Shanhe Wang, Tesfaye Getachew, Joram M. Mwacharo, Aynalem Haile, Wei Sun

**Affiliations:** 1 College of Animal Science and Technology, Yangzhou University, Yangzhou 225009, China; hty980105@163.com (T.H.); hsn138157@163.com (S.H.); shanhe12315@163.com (S.W.); 2 Joint International Research Laboratory of Agriculture and Agri-Product Safety of Ministry of Education of China, Yangzhou University, Yangzhou 225009, China; dx120170085@yzu.edu.cn; 3 International Centre for Agricultural Research in the Dry Areas, Addis Ababa 999047, Ethiopia; T.Getachew@cgiar.org (T.G.); j.mwacharo@cgiar.org (J.M.M.); a.haile@cgiar.org (A.H.)

**Keywords:** miR-143, *CUX1*, *KRT71*, wool curvature, dermal papilla cell, proliferation

## Abstract

Wool curvature is the determining factor for lambskin quality of Hu lambs. However, the molecular mechanism of wool curvature formation is not yet known. miRNA has been proved to play an important role in hair follicle development, and we have discovered a differentially expressed miRNA, miR-143, in hair follicles of different curl levels. In this study, we first examined the effects of miR-143 on the proliferation and cell cycle of dermal papilla cells using CCK8, EdU and flow cytometry and showed that miR-143 inhibited the proliferation of dermal papilla cells and slowed down the cell cycle. Bioinformatics analysis was performed to predict the target genes *KRT71* and *CUX1* of miR-143, and both two genes were expressed at significantly higher levels in small waves than in straight lambskin wool (*p* < 0.05) as detected by qPCR and Western blot (WB). Then, the target relationships between miR-143 and *KRT71* and *CUX1* were verified through the dual-luciferase assay in 293T cells. Finally, after overexpression and suppression of miR-143 in dermal papilla cells, the expression trend of *CUX1* was contrary to that of miR-143. Meanwhile, *KRT71* was not detected because *KRT71* was not expressed in dermal papilla cells. Therefore, we speculated that miR-143 can target *CUX1* to inhibit the proliferation of dermal papilla cells, while miR-143 can target *KRT71* to regulate the growth and development of hair follicles, so as to affect the development of hair follicles and ultimately affect the formation of wool curvature.

## 1. Introduction

In natural its state, a wool fiber forms a regular curl along its longitudinal axis, a characteristic commonly known as wool curvature. The degree of curvature is an important basis of evaluating the quality of wool. The growth of hair follicles in the skin causes the wool to bend, resulting in the formation of different wavy patterns of lambskin. The basic structure of wool fibers is a fundamental formation of hair morphology. The hair follicle is a specific skin appendage that has the ability to grow periodically. Traits such as the shape, type and color of wool are closely associated with the development and cycle of hair follicles. The Wnt/β-catenin, TGF-β/BMP and other signaling pathways form a complex network to control the formation and cyclic development of hair follicles, resulting in different wool wavy pattern phenotypes [1,2,3,4]. Previous research has revealed that the dermal papilla cells are the regulatory center in the development of hair follicles, and the number and type of papilla cells are decisive for the growth of hair follicles, the type and curvature of wool [5,6].

Wool curvature has been a hot topic of interest in scientific research, with increasing reports of gene and protein studies associated with wool curvature formation. So far, based on an understanding of the biological processes and molecular mechanisms of hair bending, lots of theories have been proposed to explain bending, two of which are the most accepted. A theory has explained that the hardening of ORS (out root sheath) and IRS (inner root sheath) leads to specific longitudinal or transverse shape, ultimately leading to curvature of the hair [7]. Correspondingly, in 2009, Cadieu et al. [8] reported that *KRT71* specifically expressing in IRS is a well-established curly hair gene, and Kang et al. [9] further confirmed through functional analysis that *KRT71* mRNA expression could be regulated by myeloid zinc finger 1 (MZF1) and contributes to the formation of wool curvature in Chinese lambs. Tufarelli et al. [10] detected the first cut repeat (CR1) on a mouse model, termed CUX1ΔCR1, leading to the phenotype characterized by wavy hair. Another interesting theory is that of the multiple papillary centers (MPC) model [11], in which multiple papillary centers form in each dermal papilla, and a lack of precise and coordinated growth of the papillary center is the main reason why the hair fiber is asymmetrical, suggesting that dermal papilla cells’ growth is involved in hair bending and growth. Therefore, we hypothesized that dermal papilla cells’ proliferation would have a positive effect on wool curvature.

MicroRNA (miRNA), an endogenous non-coding small molecule RNA of approximately 20–25 nucleotides in length, could cause mRNA degradation or transcriptional repression through binding to complementary regions of mRNA, which affects gene expression and thus the growth and development of animals [12]. In recent years, several studies have shown that miRNAs are effective regulators of developmental and disease processes, including those involved in animal coat growth and development, the hair follicle cycle and keratin-forming cell differentiation in the skin [13,14]. miR-203, which is called “skin-specific miRNA”, was the first miRNA found to be abundantly expressed in animals’ skin and hair follicles and is closely associated with development of hair follicles [15]. miR-205 is more highly expressed during the recession phase than the anagen phase of mouse hair follicles, driving the transition of hair follicles from anagen to recession [16]. Overexpression of miR-22 in mouse skin could inhibit the transition of hair follicles from the resting phase to the next growth phase and promote the transition of hair follicles from the growth phase to the regression phase, resulting in hair loss in mice [17]. This evidence shows that miRNAs are important regulators of the hair follicle growth cycle. In addition, miR-125b [18] and miR-203 [19] were investigated to inhibit hair follicle stem cell differentiation in mice, resulting in abnormal hair follicle stem cell proliferation and morphology, while the short-term induction of miR-22 could promote hair follicle stem cell differentiation [17]. We also found that miR-148b could promote β-catenin protein phosphorylation and the proliferation of dermal papilla cells and stromal cells, and conversely, miR-320 could inhibit the expression of dermal papilla cells and hair matrix cells [20].

In a previous study, we found significant differences in the expression of miR-143 in the small waves and large waves groups [21,22]. Simultaneously, many candidate genes were also screened, such as *BMP7*, *CDKNIC*, *POU1F1*, etc. [23]. Fortunately, *KRT71* and *CUX1* were also differentially expressed in small and large waves. Based on the MPC theory, we ventured to speculate whether miR-143 could mediate the action of *KRT71* and *CUX1* on hair follicle cells or even dermal papilla cells to regulate hair follicle growth and development leading to wool curvature. Therefore, we verified the effect of miR-143 on dermal papilla cells and then determined the expression of *KRT71* and *CUX1* in straight wool and small waves, predicted the targeting relationship between miR-143 and *KRT71*, *CUX1* by bioinformatics analysis, and demonstrated whether miR-143 could positively mediate *KRT71* and *CUX1* using the dual-luciferase reporter assay and WB. Finally, our results will provide some basis of studying the mechanism of wool curvature.

## 2. Materials and Methods

### 2.1. Skin Tissue Collection, RNA and Protein Extraction

Skin tissue samples were obtained from pre preservation in the laboratory of Livestock (Epi-) Genetics and Functional Genomics in Yangzhou University. Each sample was used for RNA and protein extraction using Trizol (TIANGEN, Beijing, China) and Total Protein Extraction Kit (Beyotime, Shanghai, China), respectively. All RNA and protein were stored at −80 °C for use. 

### 2.2. Cell Culture and Transfection

The dermal papilla cell was previously isolated and cultured from Hu lambs in our laboratory [24]. The Human embryonic kidney HEK293T was obtained from College of Veterinary Medicine at Yangzhou University. These cells were cultured in DMEM/F12 medium (HyClone, Logan, UT, USA) and supplemented with 10% fetal bovine serum (Gibco, Grand Island, NY, USA) and 1% penicillin–streptomycin (Gibco, Grand Island, NY, USA) at 37 °C under 5% CO_2_ atmosphere. The cells were grown in 6, 24, or 96-well plates to 80% confluence and transfected by using jetPRIME (Polyplus-transfection, Illkirch, France) according to the manufacturer’s instructions.

### 2.3. Cell Proliferation and Cell Cycle

To identify the function of miR-143, we transfected miR-143 mimics and inhibitors to dermal papilla cells and analyzed their effect on the proliferation of dermal papilla cells. Firstly, CCK-8 (Tecan, Shanghai, China) was used to detect the OD value (optical density value) of dermal papilla cells in 96-well plates under 450 nm at 0 h, 24 h, 48 h, 72 h after transfection with a microplate reader.

At 24 h after transfection, cell suspensions were collected to detect cell cycle in accordance with the instructions from Cell Cycle and Apoptosis Analysis Kit (Beyotime, Shanghai, China) for flow cytometric analysis (BD, Franklin Lakes, NJ, USA). At the same time, cells were stained using Cell-Light TM EdU Apollo 567 In Vitro Kit (RiboBio, Guangzhou, China), and then the images were taken by Fluorescence Inversion Microscope System (Nikon, Tokyo, Japan). 

### 2.4. Target Gene Prediction for miR-143

RNAhybrid (https://bibiserv.cebitec.uni-bielefeld.de/rnahybrid/ (accessed on 25 January 2021)) [25], miRBase (http://www.mirbase.org/ (accessed on 25 January 2021)) [26], and TargetScan (http://www.targetscan.org/mamm_31/ (accessed on 25 January 2021)) [27] were used to predict the target genes. 

### 2.5. Quantitative RT-PCR and Western Blot

After the isolation of skin tissue and cell, Quantitative RT-PCR and Western blot were performed. The first-stand cDNA was synthesized using FastKing gDNA Dispelling RT SuperMix (TIANGEN, Beijing, China), and then the mRNA expression level was detected by qRT-PCR using TB Green Premix Ex Taq II (TaKaRa, Kusatsu, Shiga, Japan). The miRNA was extracted using miRcute miRNA Isolation Kit (TIANGEN, Beijing, China) and reverse-transcribed into first-strand cDNA using miRcute miRNA First-Strand cDNA Kit (TIANGEN, Beijing, China). Finally, the miRNA expression was detected using miRcute Plus qPCR Kit (SYBP Green) (TIANGEN, Beijing, China) according to the manufacturer’s instructions. The primers’ information is listed in Table 1. *GAPDH* and U6 were used as the reference genes. Relative expression of *CUX1*, *KRT71*, *CDK2*, *PCNA*, Cyclin d1 and miR-143 were calculated using ΔΔCt method [28]. Each qRT-PCR experiment was accompanied by three biological replicates and three technical replicates.

To further identify the qRT-PCR results, Western blot was carried out. After protein extraction, the concentrations of different samples were measured by using BCA Protein Assay Kit (Beyotime, Shanghai, China). After proteins were denatured, proteins were separated by SDS-PAGE, transferred to PVDF membranes using Trans-Blot TurboTM Protein Transfer System (Bio-Rad, Hercules, CA, USA), and reprobed with 1:2000 rabbit anti-*CUX1*, 11733-1-AP (Proteintech, Chicago, IL, USA), 1:5000 rabbit anti-*KRT71*, GTX107343 (GeneTex, Shenzhen, China) and 1:3000 Mouse anti-*GAPDH*, A19056 (ABclonal, Wuhan, China) antibodies, and then with 1:2500 HRP Goat anti-rabbit (IgG), AS003 (ABclonal, Wuhan, China) antibodies. Finally, protein was reprobed with ECL chemiluminescent substrate (hypersensitive) (Biosharp, Hefei, China) for 3 min, and then detected with an analysis by ChemiDocTM Touch Imaging System (Bio-Rad, Hercules, CA, USA).

### 2.6. Luciferase Reporter Assay

The 3′UTR sequences of *KRT71* and *CUX1* containing the biding sites were cloned and synthesized to psiCHECK-2 report vector (Generay, Shanghai, China), respectively. Additionally, we designed and constructed mutant vectors using Fast Site-Directed Mutagenesis Kit (TIANGEN, Beijing, China) (Table 2). All recombinant plasmids were sequenced for accuracy by Sangon Biotech Co. Ltd. The miR-143 mimics and inhibitors were purchased from RiboBio Co., Ltd., Guangzhou, China. When the HEK293T cells were cultured in 24-well plate to about 75% confluence, the wild type, mutation type and miR-143 mimics/inhibitors were then cotransfected to the cells. After that, luciferase activity was measured using Dual-luciferase Reporter Assays (Promega, Madison, WI, USA). Our experimental groups were set as miR-143 mimics + *KRT71* or *CUX1* 3′UTR wild type (WT), miR-143 mimics-NC + *KRT71* or *CUX1* 3′UTR wild type (WT), miR-143 mimics + *KRT71* or *CUX1* 3′UTR mutation type (MUT) and miR-143 mimics-NC + *KRT71* or *CUX1* 3′UTR mutation type (MUT). All operations were carried out in strict accordance with the instructions, and each sample was set up in three parallel experiments.

### 2.7. Statistical Analysis

The differences were analyzed by independent sample t test using SPSS 16.0 software [29]. *p* < 0.05 was considered a significant difference, and *p* < 0.01 was considered an extremely significant difference. All error bars represent the means ± SE.

## 3. Results

### 3.1. miR-143 Promoting Dermal Papilla Cells Proliferation

**Figure 1:**
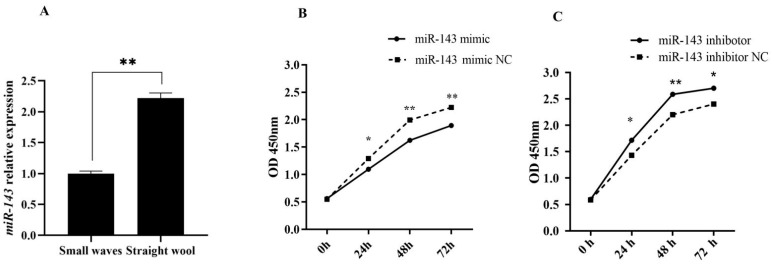
The expression level of miR-143 (**A**) in small waves and straight wool, the CCK-8 results (**B**) after transfection of the miR-143 mimic and NC, and the CCK-8 results (**C**) after transfection of the miR-143 inhibotor and NC. * *p* < 0.05 or ** *p* < 0.01.

miR-143 was found to be a significantly differentially expressed miRNA in the previous experimental results [22]. We furtherly verified that the expression level of miR-143 had a significant difference between small waves and straight wool groups (Figure 1A). Therefore, we speculated that miR-143 had a potential function in the formation of wool curvature. In order to verify the function, the effects of the overexpression or inhibition of miR-143 in dermal papilla cells on cell proliferation were detected. As ascertained from the CCK-8 results, the OD value of miR-143 mimic group was significantly lower than that of the control group at 24 h, 36 h and 72 h (*p* < 0.05). The opposite was true for the miR-143 inhibitor group, suggesting that miR-143 could suppress the proliferation of dermal papilla cells (Figure 1B,C).

**Figure 2:**
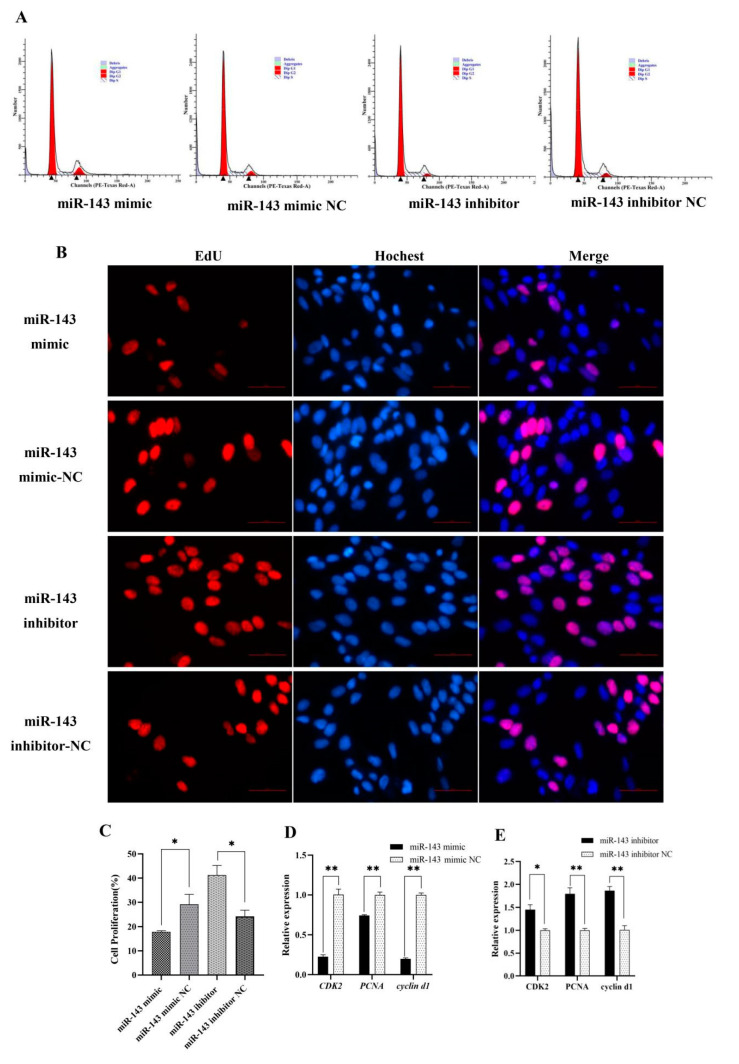
miR-143 suppressing dermal papilla cells’ proliferation: (**A**) The cell cycle assay and (**B**) EdU assay after transfection of the miR-143 mimic and inhibitor. The short red lines in each picture represent 50 μm (**C**) The count of proliferating cells. (**D**,**E**) The relative expression level of *CDK2*, *PCNA* and cyclin d1. * *p* < 0.05 or ** *p* < 0.01.

A cell cycle assay showed that the number of cells in S-phase was significantly lower in the miR-143 mimic group than in the control group, and the number of cells in G0/G1 phase was higher in the miR-143 mimic group than in the control group. The inhibitor group results were opposite to those of the mimic groups, indicating that miR-143 plays a negative role in the progression of the dermal papilla cell cycle (Figure 2A). Finally, a EdU assay and qRT-PCR further demonstrated the inhibitory effect of miR-143 on proliferation of dermal papilla cells (Figure 2B–E).

### 3.2. Target Genes Prediction of miR-143

**Figure 3:**
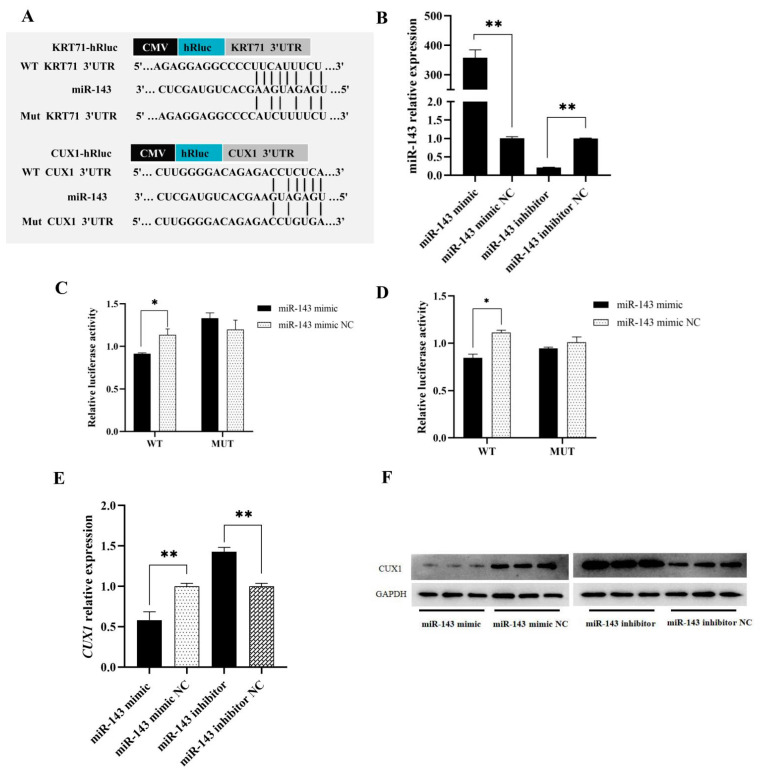
Target validation between miR-143 and *KRT71* and *CUX1*: (**A**) Target gene prediction of miR-143. (**B**) The transfection efficiency verification of miR-143 mimic and inhibitor in dermal papilla cells. (**C**,**D**) The dual-luciferase assay between miR-143 and KRT71 and miR-143 and *CUX1* in 293T cell. (**E**) Overexpression and inhibition of miR-143 in dermal papilla cells. (**F**) Detection of CUX1 protein expression level. * *p* < 0.05 or ** *p* < 0.01.

To elucidate the molecular mechanism of miR-143, bioinformatics analysis was carried out to predict their target miRNAs using miRNA hybrid, miRanda and TargetScan. The seed sequence of miR-143 was perfectly complementary to the 3′UTR of *CUX1* and *KRT71*. In addition, the expression of miR-143 in small waves was significantly lower than that in straight wool, which was opposite to that of *CUX1* and *KRT71*. Therefore, miR-143 was preferentially considered a potential target of *CUX1* and *KRT71* that needs to be validated. In particular, 294–298 bp of the *CUX1* 3′UTR and 539–548 bp of the *KRT71* 3′UTR bind to the miR-143 seed sequence (Figure 3A), respectively. 

### 3.3. CUX1 and KRT71 Highly Expressed in Small Waves Group

**Figure 4:**
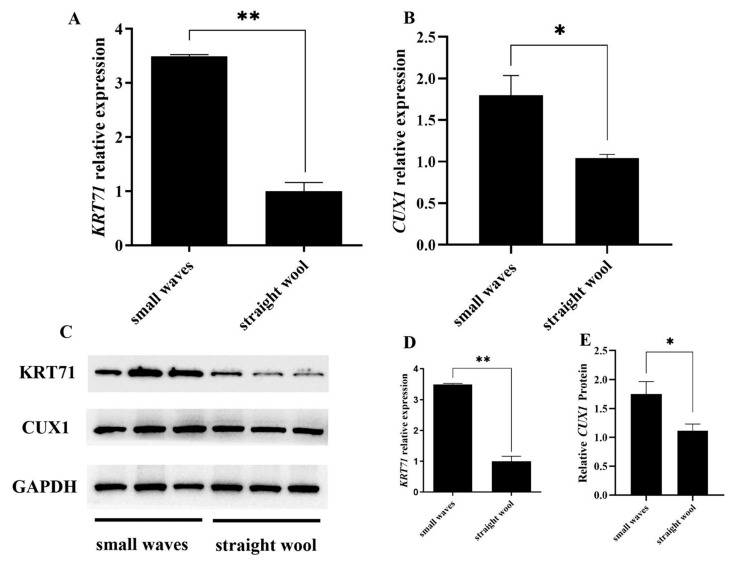
The expression levels of *CUX1* and *KRT71* in small waves and straight wool group. (**A**) The relative mRNA expression levels of *CUX1* were significantly higher in small waves group, as indicated by qRT-PCR. (**B**) The relative mRNA expression levels of *KRT71* were significantly higher in small waves group, as indicated by qRT-PCR. (**C**–**E**) The protein expression levels of *CUX1* and *KRT71* in small waves group were higher than those in straight wool, as indicated byWestern blot. * *p* < 0.05, ** *p* < 0.01.

In our study, we firstly examined the mRNA and protein expression levels of *CUX1* and *KRT71* between the small waves group and straight wool group using qRT-PCR and Western blot. The qRT-PCR results show that the mRNA expressions of *KRT71* and *CUX1* in the small waves group were significantly higher (*p* < 0.01, Figure 4A, *p* < 0.05, Figure 4B) than those in straight wool group and were consistent with the Western blot results (Figure 4C–E). The expression of miR-143 was negatively correlated with the expression of *CUX1* and *KRT71*, indicating that *CUX1* and *KRT71* were potential target genes of miR-143. These results illustrate that *CUX1* and *KRT71* might be related to wool curvature as potential makers.

### 3.4. CUX1 and KRT71 Targeting to miR-143

To further verify that miR-143 could directly target *CUX1* and *KRT71* expression, a dual-luciferase assay was performed to detect the luciferase activity after transfection for 12 h in 293T cells. The relative luciferase activity showed that no significant difference between the MUT groups (miR-143 + *KRT71* or *CUX1* 3′UTR mutation type) and control groups (*p* > 0.05), while the WT groups (miR-143 + *KRT71* or *CUX1* 3′UTR wild type) showed significantly lower luciferase activity than the control groups (*p* < 0.05) (Figure 3C,D). Therefore, miR-143 can inhibit *CUX1* and *KRT71* by binding to their 3′UTR, which suggests *CUX1* and *KRT71* are the direct target of miR-143. Then, to further clarify the relationship between miR-143 and *KRT71* and *CUX1*, we overexpressed and inhibited miR-143 in dermal papilla cells. Firstly, the effectiveness of the miR-143 mimic and inhibitor was examined using qRT-PCR, and mimic and inhibitor groups were found to be significantly different compared with the control groups (*p* < 0.01) (Figure 3B). The mRNA and protein expression of *CUX1* were also detected using qRT-PCR and Western blot, which showed that the miR-143 mimic resulted in a highly significant decrease in the expression of *CUX1* (*p* < 0.01), and miR-143 inhibitor resulted in a highly significant increase in the expression of *CUX1* (*p* < 0.01) in dermal papilla cells, revealing that the miR-143 mimic could inhibit the expression of *CUX1* protein and that the miR-143 inhibitor could increase its expression. Thus, miR-143 can reduce the expression level of *CUX1* protein, which was associated with the dual-luciferase assay results (Figure 3E,F). However, *KRT71* was not expressed in dermal papilla cells, so we speculated that miR-143 might regulate *KRT71* in other hair follicle cells to influence hair follicle development.

## 4. Discussion

Hu sheep is a unique sheep breed with white lambskin and is known world-wide for its lambskin with a waved pattern. In general, according to the pattern width and the degree of wool curvature, lambskins are divided into four types, including small waves, medium waves, large waves and straight wool, of which small waves show the best quality and straight wool shows the worst. Therefore, the molecular mechanism of wool curvature is important to reveal the molecular mechanism of lambskin pattern formation.

At present, research on hair bending is mainly conducted from a genetic point of view [30,31] and focuses on human hair [32,33,34,35,36], but wool curvature also has some common points with human hair bending, so it is worthwhile for us to learn from researche on human hair bending. Based on biology and genetics, Westgate et al. [31] proposed a valuable opinion that genetic factors and cellular processes influence the molecular mechanisms of genes and the differentiation of some hair follicle cells, determining the hair follicle morphogenesis, ultimately leading to the phenomenon of hair bending. In this theory, the proliferation and differentiation of hair follicle cells plays a positive role in the formation and maintenance of curly fibers. Genetic research has shown that various validation genes related to hair bending, including *KRT71*, *EDAR*, *TCHH*, *VDR*, *WNT10A*, etc. [9,37]. In our study, *KRT71* and *CUX1* were predicted to act as the target genes for miR-143 to explore wool curvature. *KRT71* is a famous gene that affects curly hair in dogs, and Salmela and Bauer explained that mutations in exons 2 and 7 of *KRT71* can cause curly hair in dogs [38,39]. *CUX1*, an important member of the cut family, is able to regulate cell cycle influencing protein expression as a cell cycle dependent transcription factor, and aberrantly expressed *CUX1* in a mouse model plays an important role in the cell cycle regulation of hair growth [10]. Since *KRT71* and *CUX1* are the target gene of miR-143 and have been found to be strongly associated with hair bending, it is more interesting to investigate the mechanism by which miR-143 medicates them to regulate wool curvature.

However, which part of the hair follicle structure is responsible for curly fibers and wool curvature? Some researchers have argued that curly hair originated from a hair bulb surrounded by a large number of proliferating cells, due to their uneven spatial distribution proliferating [40]. Due to the great proliferation and differential potential of dermal papilla cells, which exist in the center of the hair bulb, we explored the role of dermal papilla cells’ proliferation in wool curvature in this study.

Existing studies have proved that miR-143-5p promotes mammalian melanocyte migration and proliferation, regulates melanogenesis and is closely associated with hair color [41,42]. However, Hu sheep wool is always white regardless of whether the wool is straight or waves, so we speculate that the differential expression of miR-143 is minimally influenced by melanocytes and likely influenced by other traits, such as wool curvature. It is worth mentioning that miR-143 has also been found to be differentially expressed in hair follicles of goats during different fetal periods [43]. Apparently, miR-143 is involved in the formation of several traits in wool, but the specific regulatory mechanism is unclear. 

Currently, research on miR-143 is focused on disease, while less research has been performed on hair traits. For example, miR-143 targets *GATA6* to inhibit cell proliferation in gastric cancer cell lines and exert tumor suppression effects [44]. In the study of osteosarcoma, miR-143 directly and negatively targets *FOSL2* to inhibit osteosarcoma cell proliferation and metastasis and promote apoptosis [45]. Moreover, miR-143 could promote smooth muscle cell differentiation and inhibit cell proliferation [46]. In our results, miR-143 also had a negative effect on the proliferation of dermal papilla cells, and to some extent, could inhibit cell proliferation. 

Current studies suggest that miRNAs could be used as biomarkers. Papanota et al. found that the circulating levels of miR-143-3p, miR-17-5p, miR-214-3p and miR-335-5p were significantly higher in the blood plasma of MM patients with MMBD (multiple myeloma bone disease) compared to those without, and these miRNAs could be used as biomarkers to accurately predict MMBD [47]. Tewari et al. found that miR-10b, miR-126-5p, miR-143, miR-146b, miR-21-5p, miR-221, miR-223 and miR-30b-5p were expressed at significantly different levels in circulating skeletal muscle of Piedmontese cattle at different ages, and miR-23a and miR-126-5p could bind *Myostatin* to directly regulate the growth state of skeletal muscle [48]. In our previous studies, we found significant differences in the expression levels of miR-143, *CUX1* and *KRT71* in Hu sheep with small waves and large waves, respectively [21,22,23]. In this study, we also examined the mRNA and protein expression levels in small waves and straight wool, the findings of which were consistent with the previous results. Since the expression trend of miR-143 is opposite to that of *CUX1* and *KRT71*, we hypothesized that miR-143 might be able to mediate the role of *CUX1* and *KRT71* in hair follicle development. As far as we know, *CUX1* and *KRT71* have been reported to be involved in hair bending. Therefore, we speculated that miR-143 could be used as a biomarker for wool curvature, and these results provided a basis for further studies on miR-143-assisted lamb breeding in Hu sheep.

*CUX1* is a transcription factor that regulates the cell cycle and plays an important role in the developmental cycle of several tissues. In particular, the expression of this gene affects the cell cycle regulation of hair growth in mice, and the loss of its exon (termed *CUX1*ΔCR1) leads to hair bending [10,49,50]. To explore the function of *CUX1* in the function in vivo, Luong and Ellis separately mutated different C-terminal positions of *CUX1*. The results show that mutant mice are born with growth retardation and abnormal hair development, suggesting that *CUX1* is required for dermal developmental functions [51,52]. The keratin family has long been shown to be closely associated with hair follicle development and hair growth, and *KRT71* has also received attention as a member of this family. Fujimoto et al. [53] first discovered that a missense mutation was associated with hair loss, and identified that this gene was an important determining factor in mammalian hair growth. Previous research has shown that mutations in *KRT71* could be identified in dogs and mice; it was expressed in the inner root sheath and caused hair bending [54]. In fact, we did not specifically detect the mutations of *KRT71* in Hu sheep. *KRT71* was used as a target gene for miR-143 in this experiment, and its expression was significantly different in straight wool and small waves. However, we did not detect the protein expression of *KRT71* in dermal papilla cells, which suggests that miR-143 could not target *KRT71* to regulate dermal papilla cells’ proliferation. Therefore, we suggest that there may be another mechanism that regulates hair bending as a target gene of miRNA in other hair follicle cells.

In conclusion, this study revealed that miR-143, *KRT71* and *CUX1* were significantly differentially expressed in straight wool, and miR-143 had the opposite expression trend with both of *KRT71* and *CUX1*. Moreover, miR-143 was identified to inhibit dermal papilla cells’ proliferation. Dual-luciferase assays showed that miR-143 could directly target *KRT71* and *CUX1*. Overall, miR-143 could target CUX1 to regulate the proliferation of dermal papilla cells in Hu sheep, and the cell proliferation might lead to wool curvature, which affects the quality of the lambskin. Our findings may help us better understand the regulatory mechanism of miRNA associated with wool curvature.

## Figures and Tables

**Table 1:** The primer information of qRT-PCR.

Gene/miRNA Name	Forward Primer (5′-3′)	Reverse Primer (5′-3′)	Product Length (bp)
*CUX1*	GCACGACATTGAGACGGAG	AGCTATGGTCTCAGCCTGGT	160
*KRT71*	GGCTCATCCAGAGAATCCGC	GAGCATTGTCACCCCTCTGT	102
*GAPDH*	TCTCAAGGGCATTCTAGGCTAC	GCCGAATTCATTGTCGTACCAG	151
*CDK2*	AGAAGTGGCTGCATCACAAG	TCTCAGAATCTCCAGGGAATAG	92
*PCNA*	CGAGGGCTTCGACACTTAC	GTCTTCATTGCCAGCACATT	97
Cyclin d1	CCGAGGAGAACAAGCAGATC	GAGGGTGGGTTGGAAATG	91
miR-143	CGCGTGAGATGAAGCACTG	AGTGCAGGGTCCGAGGTATT	variable
U6	CTCGCTTCGGCAGCACA	AACGCTTCACGAATTTGCGT	95

**Table 2:** The primer for construction of vector.

Gene Name	Primer	Forward Primer (5′-3′)
*CUX1*	*CUX1*-WT-F	CCctcgagCCATCTCCCTTTAAAGAGGG
	*CUX1*-WI-R	TTgcggccgcGACTTAGAGCTCGCTGTCC
*KRT71*	*KRT71*-WT-F	CCctcgagTTGTCCCAGCTCCTGCTT
	*KRT71*-WT-R	TTgcggccgcTATTGAGGATGCAG
*CUX1*	*CUX1*-MUT1-F	CTTGGGGACAGAGACCT**G**T**G**AGCTGTGACACTG
	*CUX1*-MUT1-R	CAGTGTCACAGCT**C**A**C**AGGTCTCTGTCCCCAAG
*KRT71*	*KRT71*-MUT1-F	GGGAAGAGGAGGCCCC**A**TC**T**TTTCTCCTTTC
	*KRT71*-MUT1-R	GAAAGGAGAAA**A**GA**T**GGGGCCTCCTCTTCCC

Note: In the wild type, lowercase nucleotides indicate restriction enzyme sites. However, in the mutant type, the underlined nucleotide is the binding site for *KRT71*, *CUX1* to miR-143, and the bolded nucleotide indicates the mutation site.

## Data Availability

The raw data supporting the conclusions of this article will be made available by the authors, without undue reservation.
